# Quantifying Spatiotemporal Heterogeneity of Tumor Metabolism and Vasculature with a Multiparametric Point-of-Investigation Microscope

**DOI:** 10.34133/bmef.0207

**Published:** 2025-12-09

**Authors:** Enakshi D. Sunassee, Marcia Cunha Dos Santos, Riley J. Deutsch-Williams, Sanjana Sankholkar, Megan Madonna, Gregory Palmer, Nirmala Ramanujam

**Affiliations:** ^1^ Department of Cell Biology, Harvard Medical School, Boston, MA 02115, USA.; ^2^ Department of Biomedical Engineering, Duke University, Durham, NC, USA.; ^3^ Center for Systems Biology, Massachusetts General Hospital, Boston, MA 02114, USA.; ^4^ Department of Radiation Oncology, School of Medicine, Duke University, Durham, NC, USA.; ^5^ Department of Pharmacology and Cancer Biology, School of Medicine, Duke University, Durham, NC, USA.

## Abstract

**Objective:** The aim of this study was to develop and apply a dual-scale Capillary-Cell (CapCell) microscope to quantify spatial and temporal heterogeneity in tumor metabolism and vasculature during anti-angiogenic therapy. **Impact Statement:** This study introduces a dual-scale CapCell microscope, a novel imaging system to dynamically visualize metabolic and vascular adaptations in vivo. The platform reveals subregional features associated with treatment that are often missed by bulk analyses. **Introduction:** Tumor recurrence is often driven by microenvironmental heterogeneity in metabolism and perfusion. Given the importance of metabolic reprogramming in treatment response, the dual-scale CapCell microscope was designed to capture widefield and high-resolution images of metabolic–vascular coupling in vivo. **Methods:** The dual-scale CapCell microscope was implemented to image multiple endpoints including mitochondrial membrane potential and glucose uptake (widefield and high-resolution images) that are colocalized with vessel density and distance between vessels (high resolution). The CapCell was used to image 4T1 tumors grown in an orthotopic window chamber longitudinally following treatment with Combretastatin A-1 (CA1), a vascular-disrupting agent. Imaging was performed over a period of 8 days to evaluate the effects of CA1 administered on days 1 and 5. **Results:** Treated tumors showed a significant decrease in metabolism and vessel fraction, and a significant increase in the distance between vessels immediately following the first treatment. Within microregional areas, elevated mitochondrial activity was associated with vascular-dense regions, whereas increased glucose uptake was more apparent in less vascularized regions. Interestingly, the second treatment on day 6 had little effect on the tumor metabolism, and in fact, metabolism at this time point recovered to baseline levels despite a persistent reduction in vessel area fraction and no corresponding recovery in vascular proximity. **Conclusion:** The CapCell enables dual-scale, multiparametric imaging of tumor microenvironments, capturing spatial metabolic and vascular features often linked to poor therapeutic outcomes. This platform can inform therapeutic timing and guide the development of combination strategies by resolving critical tumor subpopulations.

## Introduction

Tumor cells are embedded within a dynamic and heterogeneous microenvironment that evolves over time and space. This heterogeneity is evident at both genotypic and phenotypic levels, influencing tumor progression and therapeutic resistance [1]. Temporal heterogeneity refers to the emergence of distinct tumor subpopulations over time, driven by evolutionary pressures such as therapy. Spatial heterogeneity, on the other hand, is characterized by phenotypic variability across different tumor regions. Both forms of heterogeneity contribute to the survival of resistant cell populations, ultimately leading to recurrence, a major cause of cancer mortality.

A central contributor to phenotypic heterogeneity is the tumor’s vascular microenvironment, which supplies nutrients and oxygen. In normal behavior, cells near functional blood vessels typically rely on oxidative phosphorylation (OXPHOS), whereas cells in hypoxic regions, farther from vasculature, preferentially shift toward glycolysis [2]. On the other hand, cancer cells do not necessarily exhibit this behavior. Aberrant angiogenesis and impaired perfusion create oxygen and nutrient availability gradients, driving selection for distinct metabolic phenotypes across the tumor. While angiogenesis can restore perfusion and support OXPHOS, tumors may also exploit angiogenic remodeling to enhance nutrient delivery and sustain rapid proliferation. In some contexts, this nutrient excess, despite oxygen availability, can reinforce aerobic glycolysis (i.e., the Warburg effect) [3–6].

Recent studies by our group and others have shown the ability of aggressive breast cancers to reprogram metabolism to achieve treatment resistance and recurrence. Dormant cells have been shown to exhibit an increased dependence on mitochondrial metabolism, confirming that metabolic flexibility to shift between glycolysis and OXPHOS is essential for tumor survival under therapeutic stress [7–9]. Our group previously demonstrated that primary tumors in a recurrent genetically engineered mouse model of HER2+ breast cancer undergo a metabolic shift from glycolysis to OXPHOS across regression, dormancy, and recurrence [10,11]. Similar patterns were observed in highly metastatic murine triple-negative breast cancer tumors but not in micro- or nonmetastatic counterparts [12]. Collectively, these studies highlight how aggressive tumors rely on metabolic reprogramming and emphasize the need to measure the key pathways supporting this cancer hallmark. Imaging serves as a powerful tool to capture spatially varying and temporally fluctuating metabolic shifts [13].

Understanding the morphology and function of tumor vasculature and metabolic activity has driven the development of several key imaging technologies. Immunohistochemistry involves the labeling of fixed tissue sections with fluorescent antibodies, serving as a reliable technique for investigating vessel architecture and distribution of ex vivo tissue by staining with an endothelial-specific anti-CD31 antibody [6]. Magnetic resonance spectroscopy allows for the quantification of tissue metabolites and, when paired with contrast-enhanced magnetic resonance imaging, can provide information on both metabolism and vasculature of bulk tissue (no single-cell resolution) [14,15]. Optical imaging leverages the unique optical absorption properties of hemoglobin to generate contrast between vasculature and surrounding tissues. Observation of vessels in this way has been demonstrated across multiple modalities including dark-field microscopy [16], laser speckle contrast imaging [17], and optical coherence tomography [18]. Photoacoustic imaging leverages the same mechanism of contrast but, owing to its ultrasound-based detection, can image across length scales and measure oxygen saturation as a surrogate marker for metabolism [19,20]. In addition to vascular imaging, optical techniques have also been widely used to study tumor metabolism. Autofluorescence of endogenous coenzymes NADH (reduced form of nicotinamide adenine dinucleotide) and FAD (flavin adenine dinucleotide) allows the calculation of the redox ratio and fluorescence lifetimes, which together indicate the balance between glycolysis and OXPHOS [21,22]. These parameters, often combined into an optical metabolic imaging index, have been utilized in patient-derived organoids to predict therapeutic responses [23–25]. Additionally, our group has demonstrated the use exogenous probes such as tetramethylrhodamine ethyl ester (TMRE) (mitochondrial membrane potential), 2-(*N*-(7-nitrobenz-2-oxa-1,3-diazol-4-yl)amino)-2-deoxyglucose (2-NBDG) (glucose uptake), and Bodipy FL C16 (fatty acid uptake) to allow direct measurement of metabolic pathways, revealing metabolic reprogramming and vulnerabilities in preclinical breast cancer models [26,27]. While all excellent modalities, there is still a lack of integrated methods for studying metabolism and vasculature in live tissue.

It is evident from previous studies that there exists a need for a system that can coimage tumor vasculature and adjacent tumor metabolism within the same field of view (FOV). The above studies also highlight the need to image both glycolysis and OXPHOS in tandem. In addition to capturing metabolic heterogeneity across a macroscopic tumor field, it would be valuable to observe high-resolution heterogeneity within local capillary-level neighborhoods. Toward this goal, we have previously developed a novel computational approach and applied it to the design of a first-generation microscope, the Capillary-Cell (CapCell), with key capabilities for quantitative fluorescence and reflectance imaging. Specifically, a computational model was used to optimize the position of a series of optical fibers in different configurations relative to each other to achieve uniform illumination for imaging at different aspect ratios (i.e., core needle biopsy, organoids, and mammary window chamber models) and resolutions (i.e., widefield or high-resolution imaging). Importantly, in all these cases, the fiber configuration concentrated all the optical illumination into the desired FOV, thus maximizing excitation power while maintaining the low-cost, portable design of this system. Uniform illumination also enables intra- and interimage analysis across all high resolution FOVs.

The goal of the current study was to extend the previously published system design, specifically for wide-field, high-resolution imaging of tumor metabolism and vasculature in vivo, and to develop a companion image analysis pipeline for studying metabolic heterogeneity arising in different vascular neighborhoods, as well as longitudinal changes in tumor metabolism and vasculature. Colocalized vessel density and distance were quantified from the high-resolution images. The capabilities of the CapCell and companion image analysis algorithms were demonstrated in an experiment in which the tumor vasculature was disrupted using an anti-angiogenic drug (Combretastatin A-1 [CA1]). A series of image analysis algorithms was developed to study the spatiotemporal relationship between vessel distribution and metabolic heterogeneity.

Treated tumors showed a significant decrease in mitochondrial metabolism and vessel fraction, and a significant increase in the distance between vessels immediately following the first treatment on day 2 (D2). Within microregional areas, elevated mitochondrial activity was associated with vascular-dense regions, whereas increased glucose uptake was more apparent in less vascularized regions. Longitudinal analysis beyond the initial suppression revealed a rebound in clusters associated with OXPHOS accompanied by increased glucose uptake. This trend was not altered despite a second administration on D5. These results suggest that pruning of vessels following anti-angiogenic therapy could have increased the availability of oxygen thereby increasing metabolism. These dynamic and spatially resolved patterns of tumor metabolism and vasculature may serve as key intervention points in future therapeutic strategies.

## Results

### Dual-scale CapCell microscope captures widefield and high-resolution images within the tumor landscape

Previously, we developed a computational model to optimize illumination geometries that allow imaging across different aspect ratios, magnifications, and FOVs. Here, we used the computational model, a global optimization algorithm, and a custom cost function to design a dual-scale CapCell for widefield and high-resolution intravital imaging of a mammary window chamber model [28]. Widefield imaging was designed to capture large-scale spatial patterns across the tumor, while high-resolution imaging would resolve microregional and cellular features. Together, they enable multiscale characterization of metabolic and vascular heterogeneity. MATLAB (Global Optimization toolbox of MATLAB 2021A [MathWorks]) was used to perform a genetic pattern search to find the optimal spatial arrangement of 4 optical fibers to uniformly concentrate all the illumination power within each FOV (widefield or high resolution). Results of the optimization process are shown in Fig. S1. The widefield geometry was used to image the fluorescence of 2 different metabolic endpoints reflecting glucose uptake and mitochondrial metabolism. The high-resolution geometry was used for both metabolic and vascular imaging.

**Fig. 1.**
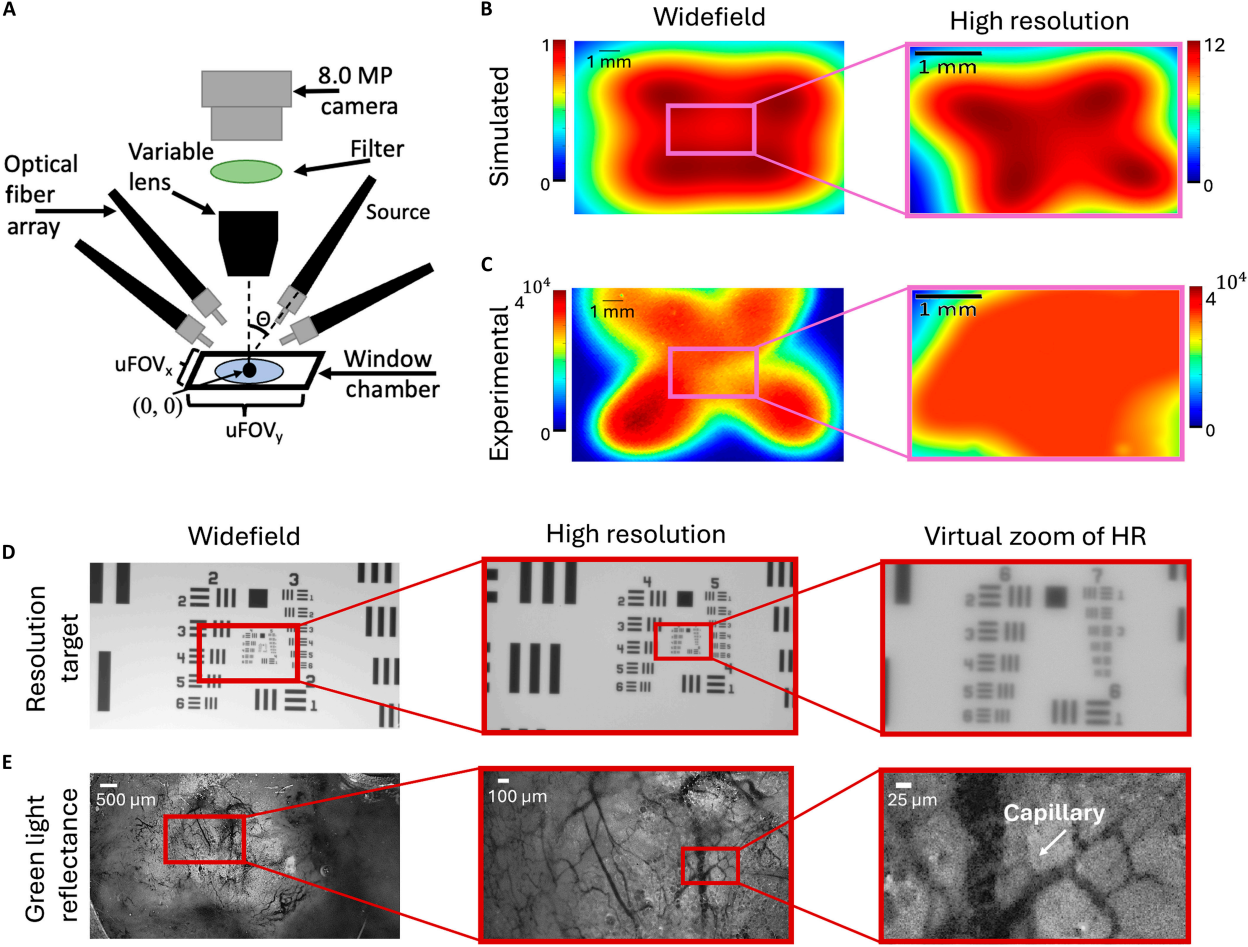
Configuration and validation of the multiscale CapCell microscope. (A) Schematic of the setup of the optical fibers, excitation filters, and LEDs. (B) Simulated and (C) experimental illumination distribution for widefield imaging (FOV is 11.2 mm × 6.3 mm) and high-resolution imaging (FOV is 2.8 mm × 1.5 mm). (D) Reflectance resolution target images captured at widefield, and high resolution, along with a virtual zoom setting. (E) Representative widefield, high-resolution, and virtual zoom images of 4T1 tumors imaged with green light, showing local vasculature. Scale bars are indicated in the figure.

The key components of the dual-mode CapCell are shown in Fig. 1A. Figure 1B and C show the simulated and experimental widefield and high-resolution images with the optimized illumination geometry, which resulted in a 12-fold increase in optical power compared to widefield imaging, due to the concentration of the light within the targeted region of interest (ROI). The resolution and FOV of the CapCell was determined using a USAF resolution target as shown in Fig. 1D. The widefield mode achieved a resolution of 13 μm and an FOV of 11.2 mm × 6.3 mm, while the high-resolution mode achieved a resolution of 5.5 μm and an FOV of 2.8 mm × 1.5 mm. To demonstrate the ability to resolve vascular structures, we used green light illumination to image 4T1 tumor-bearing mice implanted with mammary window chambers with widefield and high-resolution settings (Fig. 1E). Evaluation of the widefield images shows a large white area that corresponds to the scattering from the tumor. Though blood vessels can be seen on this white background, it is hard to resolve local vascular features. High-resolution imaging enables visualization of local vasculature in the tumor microenvironment. High-resolution images reveal individual blood vessels, all the way down to capillaries measuring 8 μm. Coupling vascular imaging with colocalized fluorescence imaging of metabolic endpoints can be used to describe the heterogeneity of glucose uptake and mitochondrial metabolism in different vascular neighborhoods.

### Treatment with an anti-angiogenic drug results in significant longitudinal changes in metabolism and metabolic heterogeneity

**Fig. 2.**
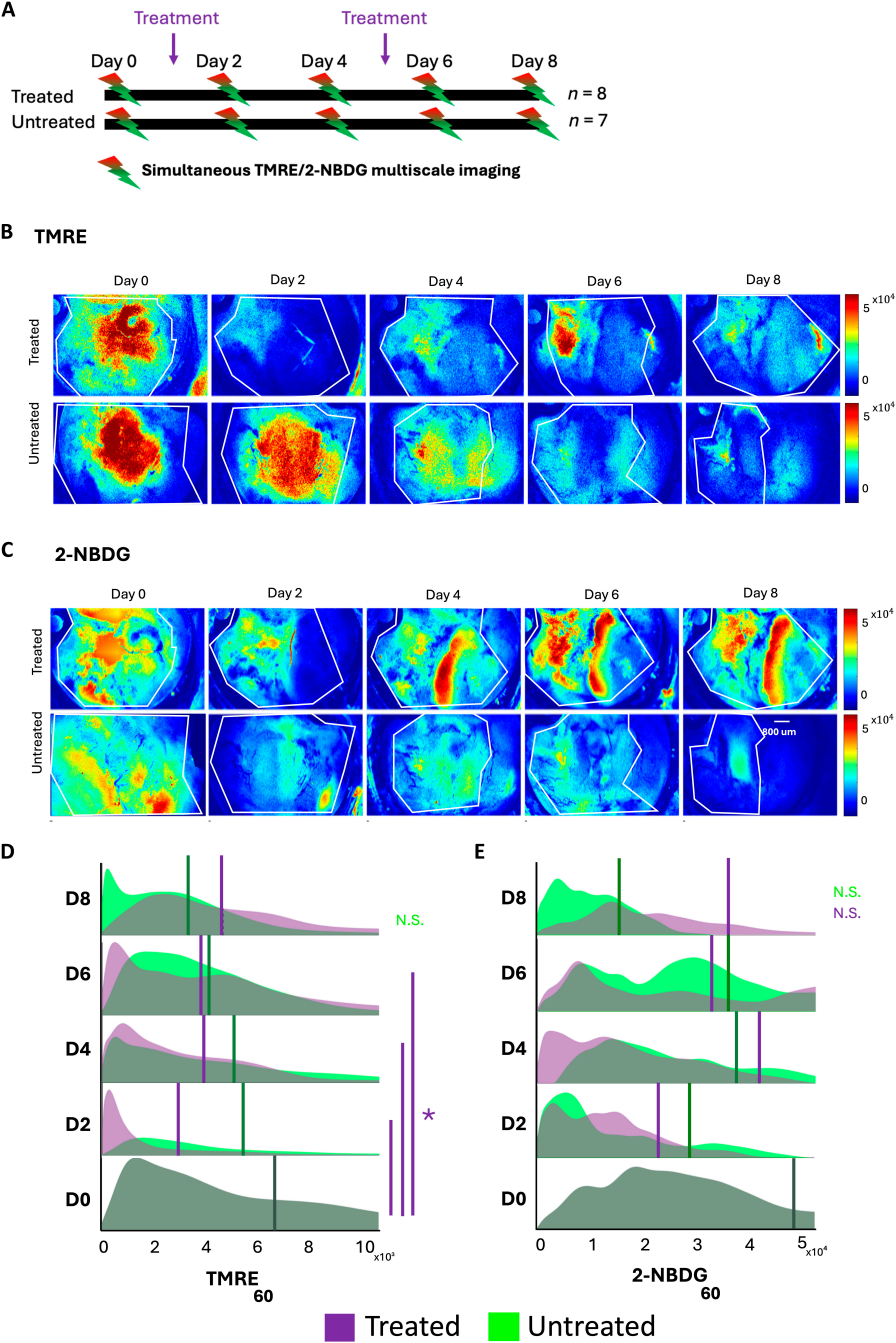
Treated tumors show longitudinal changes in bulk mitochondrial metabolism but not in glucose uptake. (A) Experimental design of the longitudinal study. Representative widefield images of treated and untreated 4T1 tumors imaged with (B) TMRE and (C) 2-NBDG at 60 min postinjection (*n* = 8 treated; *n* = 7 untreated). Scale bar and color bar are shown in the figure. Tumor regions (white outlines) were fully included in the analysis; differences across samples reflect variation in tumor size. Representative images show one mouse, whereas PDFs are pooled from all mice. Ridge plot *y*-axes vary between days but are fixed between treated and untreated groups within each day to allow direct comparison. Ridge plots showing probability density functions (PDFs) of (D) TMRE and (E) 2-NBDG for both treated and untreated tumors. Vertical dashed lines superimposed on each curve correspond to the average fluorescence. Statistical differences determined using a Kolmogorov–Smirnov test. An asterisk (“*”) signifies statistical significance between 2 time points joined by the corresponding line. White outline on widefield images corresponds to tumor regions. N.S., not significant.

First, we sought to quantify in vivo longitudinal changes in glucose uptake and mitochondrial metabolism following vascular perturbation with an anti-angiogenic agent. The 2-NBDG probe was administered 20 min after that of TMRE to eliminate biological crosstalk, a protocol that we have established previously [29]. Baseline imaging at D0 was performed when the tumors reached a volume of ~150 mm^3^. Following baseline imaging, mice were treated with a known vessel-disrupting agent, CA1 (Clement et al. 2020). CapCell imaging was performed every 2 days (D0, D2, D4, D6, and D8) (Fig. 2A). The relevant fluorescent probes were imaged at 60 min after the initial injection [10,11].

Representative widefield images of (Fig. 2B) TMRE and (Fig. 2C) 2-NBDG allow qualitative comparison of a treated and untreated tumor. The probability density functions (PDFs) (across all mice at each time point) (Fig. 2D) of TMRE and (Fig. 2E) 2-NBDG allow for a quantitative comparison. It should be noted that the D0 time point for both the treated and untreated groups corresponds to all untreated tumors combined. Statistical comparisons at D0, however, have been performed on groups corresponding to the same mice over time. There was a significant decrease in bulk mitochondrial metabolism (TMRE) in treated tumors following the first CA1 dose (D2), and significant differences from D0 persisted until D6, after which changes were no longer significant (*n* = 8 treated, *n* = 7 untreated). However, there were no significant differences in bulk glucose uptake (2-NBDG) between any of the time points within and between the 2 groups (*n* = 8 treated, *n* = 7 untreated).

**Fig. 3.**
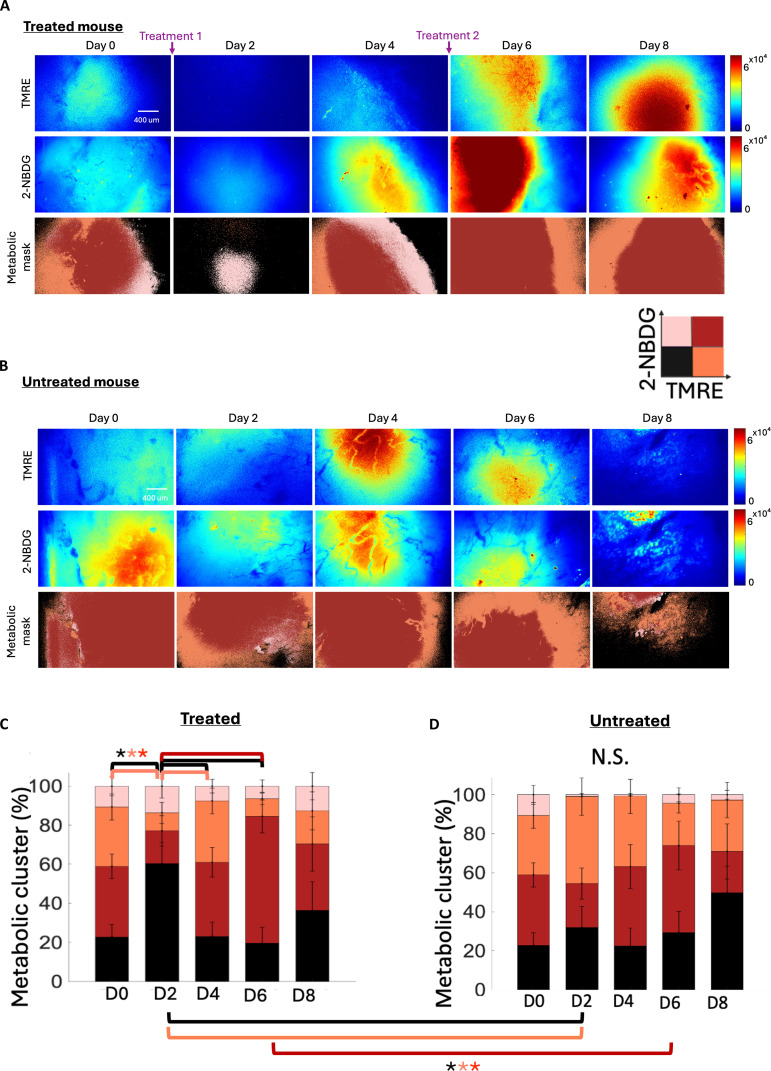
Treated and untreated tumors display longitudinal changes in metabolic heterogeneity. Representative high-resolution images of TMRE and 2-NBDG from D0 to D8 for (A) treated (*n* = 8) and (B) untreated tumors (*n* = 7). The scale bar and color bar are shown in the figure. Stacked bar graphs show the average percentage of metabolic clusters across the study for the same (C) treated and (D) untreated groups. Vertical dashed lines superimposed on each curve correspond to the average fluorescence. Statistical differences determined using a one-way ANOVA followed by Tukey’s post-hoc test. An asterisk (“*”) signifies statistical significance between 2 time points joined by the corresponding line.

Next, we sought to quantify the spatial heterogeneity of TMRE and 2-NBDG following perturbation with CA1 of the same tumors displayed in Fig. 2. High-resolution TMRE and 2-NBDG images were obtained from 3 discrete sites on the widefield metabolic image within each tumor. Metabolic cluster maps were quantified from the original 2-NBDG and TMRE images for treated (Fig. 3A and C) and untreated tumors (Fig. 3B and D), respectively. It should be noted that the representative TMRE, 2-NBDG, and the metabolic cluster map shown for the treated and untreated tumors in Fig. 3 were selected from the widefield images in Fig. 2. The 4 distinct clusters are [TMRE_High_/2-NBDG_High_] (reflecting oxidative metabolism), [TMRE_Low_/2-NBDG_High_] (reflecting glycolysis), [TMRE_Low_/2-NBDG_Low_] (low overall metabolism), and [TMRE_High_/2-NBDG_Low_] (suggesting oxidative metabolism using potentially other substrates). The distribution of the 4 [TMRE/2-NBDG] clusters in untreated 4T1 tumors remains consistent across all time points (Fig. 3B and D). In contrast, the distribution of these clusters in treated mice reflects the impact of CA1 treatment on intratumoral metabolic changes (Fig. 3A and C). The area fraction of the [TMRE_Low_/2-NBDG_Low_] cluster is significantly higher following treatment on D2 compared to baseline (D0) and late-treated time points (D4 and D6). Conversely, the area fraction of the [TMRE_High_/2-NBDG_Low_] cluster is significantly lower on D2 compared to D0 and D4 within treated mice. Taken together, these findings indicate a drastic decrease in OXPHOS immediately after treatment on D1 and a gradual shift toward increased OXPHOS after treatment underscoring the dynamic nature of the metabolic responses to treatment. The increase in [TMRE_High_/2-NBDG_High_] from D2 to D6 in treated tumors and between treated and untreated tumors on D6 reinforces this finding. The [TMRE_Low_/2-NBDG_Low_] cluster is higher, and the [TMRE_High_/2-NBDG_Low_] is lower, in treated compared to untreated mice on D2, reflecting the acute effects of treatment with CA1 within the treated group.

### Treated and untreated tumors display longitudinal changes in vasculature heterogeneity

**Fig. 4.**
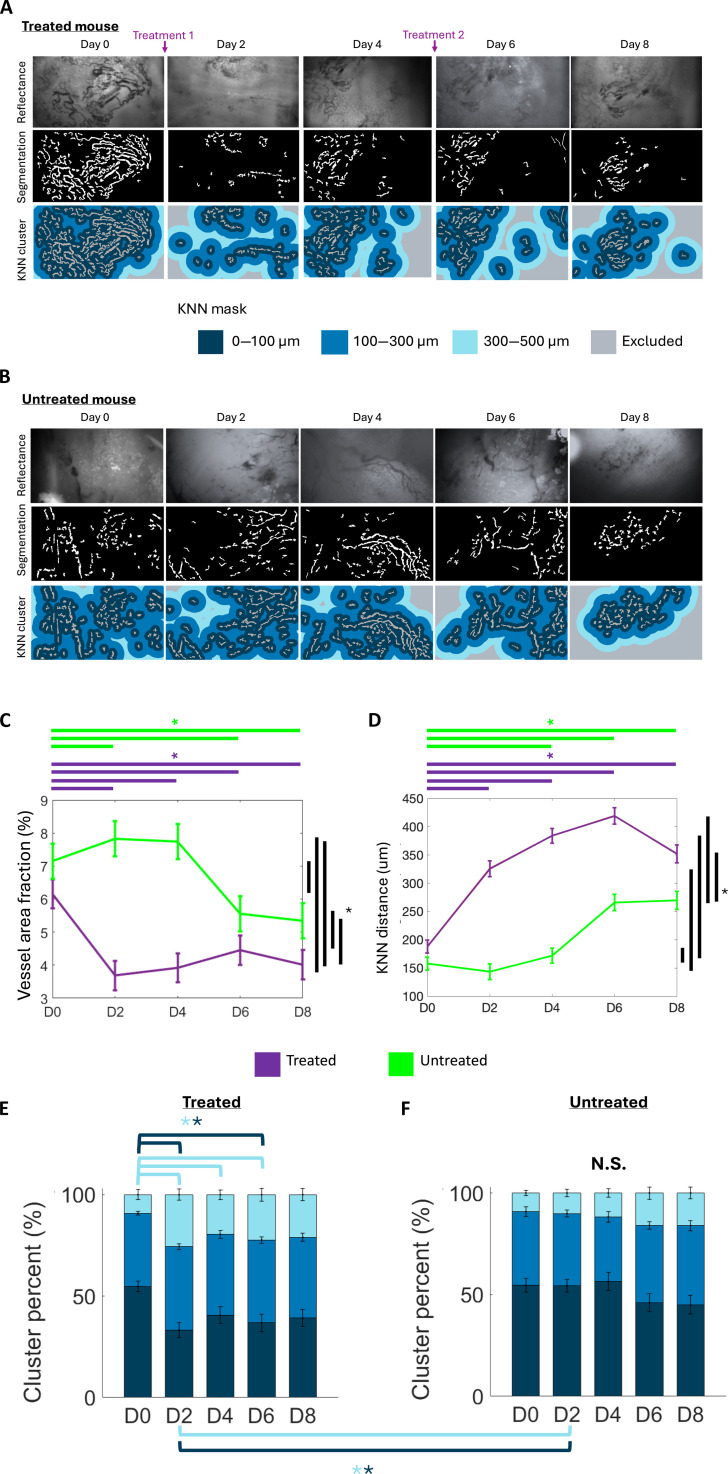
High-resolution images allow vascular heterogeneity to be compared longitudinally over the course of treatment. Representative high-resolution image and corresponding KNN map and mask of 4T1 tumors imaged longitudinally under green light for (A) treated mice (*n* = 8) and (B) and untreated mice (*n* = 7). Scale bars are shown in the figure. Plots showing the average value of (C) vessel area fraction and (D) KNN distance for each time point for treated ((*n* = 8) and untreated (*n* = 7) mice. Stacked bar graphs showing the area fraction of KNN clusters over time for (E) treated (*n* = 8) and (F) untreated (*n* = 7) mice. Statistical differences determined using a one-way ANOVA followed by Tukey’s post-hoc test. An asterisk (“*”) signifies statistical significance between 2 time points joined by the corresponding line.

We next quantified longitudinal vascular changes from high-resolution images. Qualitatively, we observed a dramatic decrease in the vessel area fraction after the first dose of CA1 on D2, which persisted until D6 (Fig. 4A). The vessel area fraction in untreated mice was comparable from D0 to D4. At D6 to D8 (Fig. 4B), however, the vessel area fraction decreased as the tumor grew, as shown by the green light reflectance images and their corresponding masks, indicating more sparse vessels in later time points. A quantitative evaluation showed that the vessel fraction decreased significantly in treated tumors (Fig. 4C) and plateaued after D6, showing that the tumors are minimally impacted by the second CA1 treatment (Fig. 4C). Untreated tumors showed minimal changes until D8, likely due to the increasing size of the tumors.

We next sought to quantify the effects of CA1 treatment on the distribution of the K-nearest neighbor (KNN) distance clusters to report on the distance between tumor tissue and its nearest vessel. Qualitatively, Fig. 4A shows a reduction in vessel fraction and a concomitant increase in the distance between vessels in treated tumors. This is reflected in the KNN cluster map as a decrease in the number of pixels with distance from vessels within the 0 to 100 μm range. Figure 4D shows that the KNN distance increased by 2-fold between D2 and D6. Further, a significantly lower percentage of clusters corresponding to a KNN distance of 0 to 100 μm and a significantly higher percentage of clusters corresponding to 300 to 500 μm were observed in treated compared to untreated mice on D2 (Fig. 4E and F). Within the treated group, we observed a significant decrease in the KNN distance of 0 to 100 μm on D2 and D6 compared to D0 and a significant increase in KNN distance of 300 to 500 μm on D2, D4, and D6 relative to D0. Over time, untreated mice showed no significant changes in the cluster contributions. These findings reflect a progressive and spatially dependent remodeling of the tumor vasculature.

### Metabolic fluctuations are a function of the vascular landscape of the tumor microenvironment

**Fig. 5.**
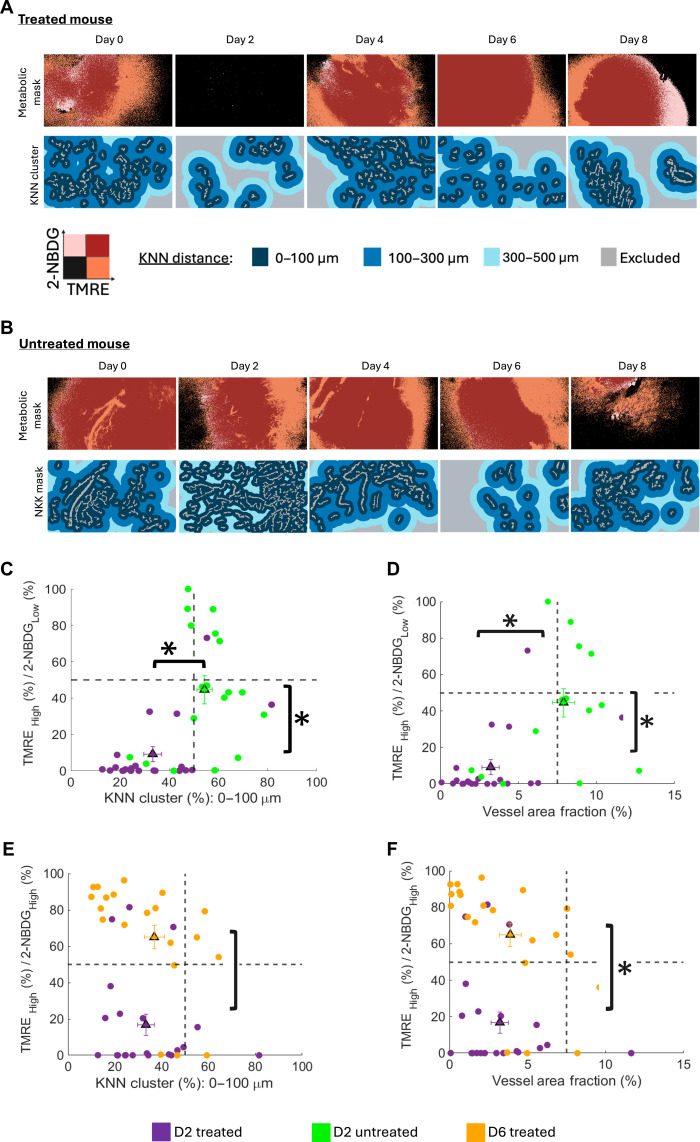
Cluster maps show the spatial relationship between metabolic and vascular heterogeneity. Representative metabolic clusters of TMRE/2-NBDG and the corresponding KNN mask of (A) treated (*n* = 8) and (B) untreated tumors (*n* = 7). Scale bars are shown in the figure. Scatter plots showing (C) the 0 to 100 μm KNN distance and (D) mean vessel area fraction vs. metabolic clusters on D2 (*n* = 8 treated; *n* = 7 untreated). Scatter plots showing (E) the 0 to 100 μm KNN distance and (F) mean vessel area fraction vs. metabolic clusters on D2 and D6 (*n* = 8 treated; *n* = 7 untreated). In the scatter plots, each dot represents the percentage of a specific metabolic or vascular cluster within a single high-resolution image, with dots color-coded by treatment condition or days in a defined treatment. Statistical differences determined using a one-way ANOVA followed by Tukey’s post-hoc test. An asterisk (“*”) signifies statistical significance between 2 time points joined by the corresponding line.

Representative images, selected from the full cohort of animals (treated, *n* = 8; untreated, *n* = 7), show the corresponding metabolic and vascular/KNN masks for each group (Fig. 5A and B) between D0 and D8. The representative images qualitatively reveal distinct spatial relationships between metabolic activity and vascular distribution in treated and untreated tumors, as well as between treated tumors at specific time points (Fig. 5A and B). Treated mice show a marked reduction in [TMRE_High_/2-NBDG_Low_], the KNN distance of 0 to 100 μm, and vessel fraction, reflecting a shift toward regions of lower mitochondrial activity with treatment (Fig. 5C and D). In contrast, in untreated mice, the metabolic cluster [TMRE_High_/2-NBDG_Low_] is higher near dense vascular regions in the 0 to 100 μm range, indicating preserved coupling between mitochondrial activity and vascular proximity in untreated tumors (Fig. 5C and D). There is rapid spatial disruption of metabolic–vascular coupling in treated tumors (Fig. 5C and D). Moreover, when considering the full landscape of all samples (Fig. 5C and D), both treated and untreated, a trend is apparent in which tissue regions with higher vascular density are enriched for the [TMRE_High_/2-NBDG_Low_] clusters. This suggests that under conditions of low glucose uptake, mitochondrial metabolism is elevated in proximity to vascular-dense regions, further supporting the association between vascular proximity and increased mitochondrial activity.

Another interesting finding is that when comparing D2 and D6, days that are post-first and -second CA1 treatment, respectively, [TMRE_High_/2-NBDG_High_] is significantly higher in D6 compared to D2 (Fig. 5E and F). This points toward a potential reversal toward oxidative metabolism and an increased reliance on glucose at later time points (D6), which appears to be independent of the 0 to 100 μm KNN cluster contribution or vessel area fraction. These findings suggest a possible temporal decoupling between vascular proximity and oxidative metabolic activity, raising the possibility that metabolic reprogramming may persist or rebound even without clear evidence of vascular normalization. Together, these results underscore the utility of the dual-scale CapCell for capturing dynamic, spatially resolved shifts in tumor metabolism in response to therapy.

## Discussion

This work presents a novel imaging platform that combines advances in optical system design with image segmentation and analysis algorithms. This platform provides a previously unattainable snapshot of the interaction between tumor microvasculature and metabolic activity. We leverage a well-established vascular-disrupting agent (VDA), CA1, as a model system to demonstrate the ability of our system to observe simultaneous changes in vascular features and metabolic activity in an in vivo tumor model. Additionally, the simplicity of the imaging hardware makes this platform adaptable for use in various laboratory and clinical settings. We demonstrated that the CapCell imaging platform can quantify spatiotemporal metabolic heterogeneity in vivo across 2 spatial scales (widefield and high resolution). Widefield images show the entire landscape and allow for the selection of local regions to image at high resolution. High-resolution images showed local variations in metabolism and vascular morphology. Another important feature of the CapCell is that it concentrates all the illumination power into a specific FOV, thereby improving the power budget for high-resolution imaging by 12-fold. This is particularly important to improve contrast from the relatively weak fluorescence of the metabolic fluorophore TMRE. Furthermore, optimizing the system to achieve uniform illumination across the entire tumor enabled the comparison of metabolism between high-resolution images within a tumor and between tumors.

Cells in closer proximity to the vasculature have long been described as exhibiting elevated mitochondrial activity. In contrast, distant cells display increased glucose uptake, consistent with the well-established metabolic response to hypoxia driven by HIF-1 activation [30]. In our dataset, this relationship is supported by the trend observed across all samples (Fig. 5D), where regions with higher vascular density were enriched for [TMRE_High_/2-NBDG_Low_] clusters, indicating elevated mitochondrial metabolism in vascular-proximal regions in conditions of low glucose uptake. Following the first treatment on D2, the tumors exhibited decreased metabolism and increased intervessel distance, whereas the control tumors showed the opposite, as expected. However, beyond this initial suppression, there was a rebound in clusters associated with oxidative metabolism, pointing to a temporal divergence between intervessel distance and mitochondrial activity. Further, the second treatment on D5 had little effect on this trend. Given that vascular disrupting agents have been shown to increase oxygen concentration at the treatment site [31], the pruning of the vasculature by CA1 may contribute to the observed increase in oxidative metabolism. Incorporating measurements of hemoglobin oxygenation into the current system would help confirm these findings.

In future studies, it would be beneficial to include the addition of Bodipy FL C16 to better evaluate the role of nutrients derived from lipids following treatments with anti-angiogenic agents. Prior studies have shown that tumor cells can boost oxidative metabolism during periods of nutrient deprivation or therapeutic stress by relying on fatty acid oxidation to sustain their energy due to a lipid-rich microenvironment [32]. In fact, our previous publications have revealed that treatment with fatty acid inhibitors can reverse the increased mitochondrial activity seen in treatment-resistant tumors. Additionally, we have demonstrated the use of the fluorophore Bodipy FL C16, showing that it reports on fatty acid metabolism in breast cancer [27] and can be integrated with our other metabolic probes [33].

It is important to note that the CapCell is not limited to TMRE and 2-NBDG imaging. Other fluorophores can be used (for example, 600 to 700 nm) that do not overlap with TMRE or 2-NBDG, allowing for the identification of specific cell types. This could enable the study of the relationship between tumor cells and other cells in the microenvironment. For example, glucose uptake and mitochondrial metabolism could be studied in the context of the immune cells, specifically CD8^+^ T cells in transgenic C57BL/6 and BALB/c mice expressing a far-red fluorescent protein, mCardinal [34]. This could allow for insight into tumor–immune cell metabolic competition in different vascular neighborhoods, an important factor in T-cell activity [16,35,36], or to study the metabolic activity of cancer-associated fibroblasts, which have been shown to drive angiogenesis [37,38].

## Materials and Methods

### Design of the dual-scale microscope

The dual-scale CapCell system was developed to enhance resolution and sensitivity compared to a previously developed system [28]. The improvement in resolution and FOV was achieved by updating the camera to an 8-megapixel monochrome complementary metal-oxide-semiconductor (CMOS) camera (Basler AG) with a variable magnification lens (Infinity Photo-Optical). The lens tube length was adjusted by 65 mm to transition between widefield and high-resolution imaging. To deliver excitation light for the proposed fluorescent endpoints (TMRE and 2-NBDG), a 1:4 fan-out optical fiber (Thorlabs) was coupled to the system. 2-NBDG was excited with a 470-nm light-emitting diode (LED) and a 460 ± 15 nm excitation filter. TMRE was excited with a 565-nm LED and a 540 ± 25 nm excitation filter. This same illumination was used to measure hemoglobin absorption with no filter in the emission path. Fluorescence emissions for 2-NBDG and TMRE were collected with a 535 ± 21 nm emission filter and a 592 ± 24 nm emission filter, respectively. A commercial translation stage and a 3-dimensional printed stage completed the system by holding the optical fibers and camera together.

To capture widefield images, a low aspect ratio illumination profile was implemented based on a previous validated study [28]. For the higher-resolution FOV, the illumination profile was optimized to maximize contrast using a previously established computational model and global optimizer. Briefly, the light emitted by each optical fiber, positioned spherically above the sample, was modeled computationally as a Lambertian source with empirically measured half-angle [28]. The Optimization Toolbox in MATLAB (MathWorks) was used to perform a genetic pattern search optimization to determine the optimal spatial arrangement needed to achieve uniform illumination with the 4-fiber system, as shown in Fig. S1. An optimization was performed to ensure uniform illumination for an FOV of 2 × 2.5 mm. For up to 50,000 iterations, with a mesh and step tolerance of 1 × 10^−6^, the optimization considered 5 spatial variables for each individual fiber that were optimized: radial distance from source to sample (*R*), angle between source and positive *z*-axis (𝜃), angle between source and positive *z*-axis (𝜃), angle between source and positive *x*-axis (ɸ), and a shift in the center point of the source illumination (d*x*, d*y*).

### Animal models

All animal procedures were approved by the Duke University Institutional Animal Care and Use Committee under protocol number A038-21-02. The animals were housed in an on-site facility with unrestricted food and water access and maintained on a standard 12-h light/dark cycle. During all optical imaging studies, the animals were anesthetized with isoflurane administered via inhalation, using a mixture of 1.5% isoflurane gas in oxygen. The murine mammary carcinoma cell line (4T1) was used to generate orthotopic mammary tumors for all mammary window imaging experiments. These cells, sourced from the American Type Culture Collection, were cultured in 90% RPMI-1640 medium (L-glutamine), 10% fetal bovine serum, and 1% penicillin–streptomycin under sterile conditions at 37 °C and 5% CO_2_. All cell cultures were maintained within 10 passages. For the experiments, 50,000 4T1 cells per mouse were injected into the fourth right mammary gland of 6- to 8-week BALB/c mice (Charles River, Raleigh, NC) with a body weight of 20 to 25 g. Once tumors grew to approximately 5 × 5 mm, the skin was removed and replaced with a 12-mm titanium window and a No. 2 glass coverslip. Mice were given at least 24 h to recover from surgery before imaging, and window chambers were monitored for up to 2 weeks or until the humane tumor burden limit was reached.

### In vivo imaging

Metabolic imaging was conducted using exogenous fluorescent contrast agents. TMRE (Life Technologies/Thermo Fisher Scientific) was used to measure mitochondrial membrane potential, and 2-NBDG (Thermo Fisher Scientific) was used to monitor glucose uptake. All mice were fasted for at least 2 h before imaging (with water provided) to ensure a normalized metabolic rate, as previously established [39]. Fasting was confirmed by measuring blood glucose levels using glucose test strips (Abbott, Alameda, CA, USA). Mice were anesthetized using 2% v/v isoflurane for induction and maintained at 1% to 1.5% v/v isoflurane during the imaging procedure. Background green light reflectance and fluorescence images were captured using widefield imaging settings. For green light imaging, an integration time of 15 ms was used, while both fluorescence channels used an integration time of 1,000 ms. Camera gain was set to 16 for 2-NBDG and 24 for TMRE during widefield imaging. In high-resolution imaging, integration times remained the same as in widefield, but the system gain was adjusted: 16 for 2-NBDG and 36 for TMRE. After background imaging, animals received a staggered injection of 750 μM TMRE followed by 6 mM 2-NBDG after 15 min. Sixty minutes post-initial injection, widefield fluorescence and green light reflectance images were acquired, and 3 high-resolution image sets were collected across the tumor. The regions were selected to ensure coverage of different quadrants within the larger image and to account for 30% of the area of the larger FOV. This approach allowed the assessment of heterogeneity across diverse intratumoral microenvironments while minimizing selection bias.

### CA1 treatment

Animals were treated with CA1, a known VDA [39], to evaluate the capability of the CapCell to capture intratumoral changes in vasculature and metabolism. After pretreatment imaging, the animals received CA1 (MedChemExpress), which was dissolved in a 20% w/v solution of SBE-β-CD (sulfobutylether-β-cyclodextrin) (MedChemExpress) in saline to improve solubility. On the day of treatment, the treated group (*n* = 8) was administered 25 mg/kg of CA1 via intraperitoneal injection of a 100-μl bolus. A control group of untreated mice (*n* = 8) was imaged longitudinally. CA1 was administered on both D1 and D5. Metabolic imaging was carried out at 12 h posttreatment for each dose.

### Analysis of widefield and high-resolution metabolic images

MATLAB (MathWorks, USA) was used for all postprocessing image analysis. To account for daily light source variations, all images were background subtracted. Ridge plots were chosen to display how the fluorescence intensity distribution changes across entire images over time for each metabolic endpoint. *y*-axis scales were allowed to vary between days but were fixed between treated and untreated groups within each day to enable direct comparison. To assess changes in metabolic probe uptake, we used a Kolmogorov–Smirnov statistical test with blocked permutations. This involved performing 1,000 random permutations per test to compare distributions before binning the data for graphing [40]. For demonstration purposes, metabolic changes over time were compared relative to treated mice, with D0 representing untreated tumors in both groups. Importantly, statistical comparisons at D0 were performed within the same mice over time.

The spatial metabolic heterogeneity of mitochondrial metabolism and glucose uptake were analyzed as described in a previous publication [41]. Three high-resolution images per animal (*n* = 8 treated; *n* = 7 untreated per probe study) were analyzed to highlight local features across different areas within the widefield FOV. For each pair of endpoints, 4 metabolic clusters were quantified: [TMRE_Low_/2-NBDG_Low_], [TMRE_High_/2-NBDG_High_], [TMRE_High_/2-NBDG_Low_], and [TMRE_Low_/2-NBDG_High_]. The abbreviated nomenclature indicates the metabolic endpoint followed by a subtext indicating whether it is high or low. Cluster assignment was performed pixel-by-pixel on all images corresponding to all mice at each time point for treated and untreated groups. The mean values of each metabolic indicator were used as cutoffs for segmentation into “high” and “low” clusters. We computed the area fraction of each cluster within each image in each group. The area fraction or cluster distribution was calculated as a percentage and area fractions were compared across time points and cell lines using a one-way analysis of variance (ANOVA) followed by Tukey’s post-hoc test for multiple comparisons.

### Vessel segmentation and vascular heterogeneity analysis

Given that capillaries are the site of nutrient exchange, only high-resolution images in which capillaries are resolvable were considered for this analysis. Vessel segmentation was conducted on green light images using a previously validated method combining Gabor filtering and Dijkstra-based segmentation [42,43]. Several preprocessing steps were introduced to enhance segmentation accuracy. Images were first processed with a median filter, followed by adaptive histogram equalization to improve local contrast. To account for background brightness variations caused by metabolic gradients, the original image was divided by a Gaussian-filtered version (*σ* = 100). Images were resized to 540 × 960 pixels to optimize segmentation run-time. A Gabor filter bank was generated with 10 orientations evenly spaced between 0° and 179° and 3 scales (*σ* = 0.3, 0.5, and 0.75). After Gabor filtering, Dijkstra forest-based segmentation was performed. As a postprocessing step, spurious edge pixels and small pixel clusters with an area of less than 50 pixels were removed.

Vessel density was determined by calculating the ratio of foreground (vessel) pixels to background pixels. To assess spatial relationships between tissue and vasculature, a KNN distance map was generated for each image. Each pixel not classified as vasculature was assigned a distance value to its nearest vessel. These KNN distances were then categorized into 3 biologically relevant bins: 0 to 100 μm, 100 to 300 μm, and 300 to 500 μm, based on known limits of oxygen and glucose diffusion [44–46]. Pixels within each bin were used to create binary cluster masks, enabling the spatial mapping of tumor regions in relation to vascular proximity. Quantitative metrics of vascular heterogeneity included vessel area fraction (ratio of segmented vessel pixels to total tissue area) and KNN distance distributions. These metrics were calculated for treated and untreated groups across all time points. Statistical comparisons of vessel density and KNN distance were conducted using a one-way ANOVA followed by a Tukey’s post-hoc test.

## Data Availability

The data that support the findings of this study are available from the corresponding author upon reasonable request and through collaborative investigations.
