# Supplementary figures and images for: Quantifying Spatiotemporal Heterogeneity of Tumor Metabolism and Vasculature with a Multiparametric Point-of-Investigation Microscope
Source: BME Front. 2025 Dec 9;6:0207. doi: 10.34133/bmef.0207 (PMC12688417; doi:10.34133/bmef.0207)

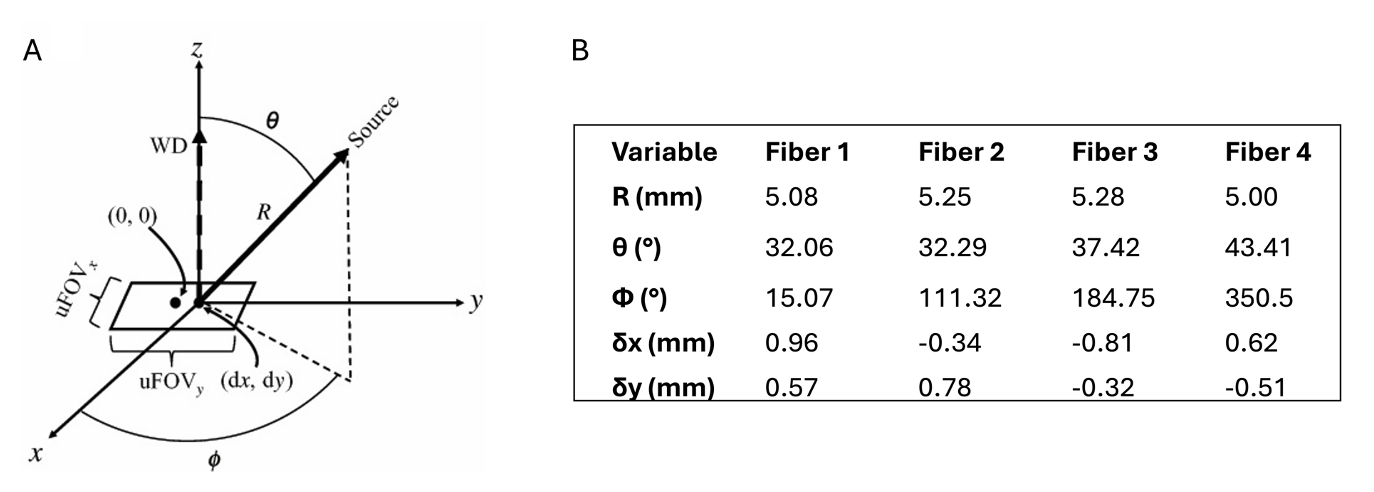

Supplement: Supplementary 1 — Fig. S1 [file bmef.0207.f1.zip › Capcell_SupFig1.png]
